# Quality assessment and nutrient uptake and utilization in Luohan pine (*Podocarpus macrophyllus*) seedlings raised by chitosan spraying in varied LED spectra

**DOI:** 10.1371/journal.pone.0267632

**Published:** 2022-04-28

**Authors:** Qiyan Song, Liang Xu, Wei Long, Jia Guo, Xie Zhang

**Affiliations:** 1 Institute of Forest Protection, Zhejiang Academy of Forestry, Hangzhou, China; 2 Forest Food Research Institute, Zhejiang Academy of Forestry, Hangzhou, China; 3 Research Institute of Subtropical Forestry, Chinese Academy of Forestry, Hangzhou, China; 4 Chengbang Eco-Environment Inc., Hangzhou, China; 5 Institute of Botany, Hunan Academy of Forestry, Changsha, China; Department of Agronomy, University of Agriculture, Faisalabad, PAKISTAN

## Abstract

Target seedling cultivation pursues high quality and nutrient utilization instead of increasing growth and size. Exposure to light-emitting diode (LED) spectra is a well-known approach that can accelerate growing speed in tree seedlings, but it is still unknown whether seedling quality and nutrient utilization would be further improved with exogeneous polymer additives. Luohan pine (*Podocarpus macrophyllus*) seedlings were exposed to red (red-green-blue lights, 71.7%-13.7%-14.6%), green (26.2%-56.4%-17.4%), and blue (17.8%-33.7%-48.5%) LED-light spectra with half receiving leaf spray by chitosan oligosaccharides (Cos) at a rate of 2 ppm (w/w) and the other half receiving only water. The red-light spectrum promoted height, biomass, nutrient utilization, and quality assessment (*DQI*) in water-sprayed seedlings. The Cos spray enhanced fine-root growth, protein, and chlorophyll-b contents with elevated nutrient utilization and quality in seedlings in the green-light spectrum. *DQI* was found to have a positive relationship with phosphorus utilization. In conclusion, although the red-light LED spectrum can promote seedling growth, green light combined with Cos spray is recommended with the aim of maintaining seedling quality and increasing P utilization in Luohan pine seedlings.

## Introduction

The ‘target plant concept’ is widely used as a guide for nursery culture of tree stocks to meet the aim of successful restoration in harsh sites. Morphological and biological attributes in seedlings are modified to overcome or acclimate to field stressors [1, 2]. Target seedlings are also necessary for successful restoration of urban forests, where tree species with a high utilization value in landscapes are frequently needed [3]. Quality assessment is an easy and applicable approach to evaluate the success for the culture of target seedlings [4]. To produce high-quality seedlings for slowly growing species, significant costs of time expense, land use, and labor investment are needed during nursery culture. Alternative approaches to promote quality of slowly growing seedlings is always favored for target seedling projects.

Evidence that tree growth speed is promoted by daily photoperiod is accumulating across species [5–7]. Recent studies have been concerned about the quality of seedlings exposed to light-emitting diode (LED) spectra [6, 8–11]. LED lighting can supply seedlings with a desired spectrum comprising different proportions of photosynthetic photon flux density (PPFD) in wavelengths of red-, green-, and blue-lights (600–700 nm, 500–600 nm, and 400–500 nm, respectively) [12–14]. The red-light spectrum induces an effect on seedling growth which was mostly the promotion on height growth [8, 11, 12, 15]. The blue-light spectrum yielded more growth and dry mass accumulation [11, 16]. The green-light spectrum at low blue-light PPFD can also promote growth in slowly growing species [6, 13]. However, seedling quality promotion may depend more heavily on the interaction between lighting and additional manipulations rather than the unique use of a spectrum.

Inherent nutrient status is the key attribute to assess quality of seedlings in restoration projects [17]. LED Spectra were examined concerning the generation of probable tradeoff between enhanced growth and diluted nutrient reserve [18, 19]. In LED lighting spectra, nutrient dilution likely occurs if no additional nutrients are added [20, 21]. A spectrum that can benefit seedling growth can still result in nutrient dilution even at a high dose of delivery [6, 19, 20]. Therefore, additional manipulation rather than nutrient supply is needed to promote nutrient uptake and utilization under LED lighting.

Chitin is a typical polysaccharide which consists of N-acetyl D-gluCosamine repeat blocks joined with β-1,4 glyCosidic bonds [22]. Chitins can be converted to derivatives, which are widely known as “chitosan” [23]. Chitosan oligosaccharide (Cos) is the final product of chitosan through enzymatic hydrolyzation [24]. Cos has an effect to trigger the tissue-specific immunity by up-regulating the reactivity of reactive oxygen species and a series of consequent physiological activities [25]. Citation added. Cos addition can promote seedling quality and nutrient uptake and utilization in silvicultural projects of tree seedlings [14, 26, 27]. Cos addition can also induce a strong resistance to abiotic stress and moderate biomass allocation to roots [14, 27, 28]. Cos addition can also interact with the LED spectrum to form a stronger promotion on seedling quality [14, 29]. All these findings suggest it is necessary to continue tests of Cos addition on quality and nutrient cycling in slowly growing seedlings cultured under continuous LED spectra.

Luohan pine (*Podocarpus macrophyllus*) belongs to the Podocarpaceae family which is widely used in urban greening and potted gardening projects in tropical and sub-tropical cities [30]. Luohan pine seedlings have a slow growing speed, hence they are frequently cultured under continuous lighting with intensive nutrient loading to quickly promote quality [31–33]. Luohan pine seedlings have been found to respond to Cos spray by enhancing resistance to drought [27]. Therefore, Luohan pine is ideal to test the combined effects of spectral condition and Cos spray on seedling quality and nutrient utilization. In the present study, we raised Luohan pine seedlings using three typical lighting spectra with Cos spray. Our objective was to detect the nutrient uptake and utilization of Luohan pine seedlings subjected to both Cos addition and different spectral conditions. We also aimed to reveal relationships between nutrient uptake and utilization in Luohan pine seedlings under Cos sprayings and LED illumination. We hypothesized that: (i) the optimum seedling quality and nutrient utilization can be obtained with Cos spray and blue light spectrum, and (ii) nutrient utilization will have a positive relationship with the enhancement of nutrient uptake. The first hypothesis was concerned by consideration of promoted growth and biomass through blue light exposure [11, 16] and the allocation of dry mass to roots induced by Cos may further strengthen whole-plant quality [14, 27, 28]. The second was put forward based on empirical findings about synchronized nutrient uptake and utilization [18, 21, 30, 34].

## Materials and methods

### Study condition and plant materials

We collected Luohan pine seeds from mother trees in Hangzhou (30°10’ N, 120°20’ E) and sterilized seeds as described by Xu et al. [31]. Half of the seeds were soaked in Cos solution (GlycoBio Inc., Dalian, China) at an immersing rate of 2 mg kg^−1^ (w/w) [27] with the other half in distilled water, both were soaked for 12 h. Seeds were sown to sands in a growing chamber at a belowground depth of 0.5 cm, moisture was maintained at 80% and temperature was controlled at 22°C. The indoor environment eliminated sunlight with all illumination supplied by artificial lighting at a condition fulfilling plant growth (PPFD in a range between 90 and 100 μmol m^-2^ s^-1^). No significant air flow was allowed around seedlings to an extent that would impact their growth.

Germinant plantlets were cultured at the juvenile stage for two months and transplanted to plastic pots (7.5cm top Ø, 7.0 cm bottom Ø, and 7.0 cm depth). Initial seedling growth at time of transplant was measured as height (4.0 cm) and root-collar diameter (RCD) (1.4 mm). These morphologies were also employed in other studies as the basic requirement for transplanted Luohan pine seedlings [27, 30, 32]. Seedlings were planted in commercial growing media (55% peat, 20% spent-mushroom residue, and 25% perlite, v/v/v) (Zhiluntuowei Inc., Changchun, China). The chemical composition of initial substrates can be found in Xu et al. [31].

### Spctra and Cos treatments

Seedlings were watered by a sub-irrigation system once a week using a 3–5 mm of water table for Luohan pine seedlings. Twelve pots of seedlings were grouped in a tank (55.5 cm length, 36.5 cm width, and 7 cm depth). Seedlings that had been previously coated using Cos solution during their seed phase were sprayed with Cos on needlelike leaves every week. The other half which was soaked in water only received water spray. Fertilizers were applied to seedlings at a rate of 60 mg N plant^−1^ according to an exponential fertilization model [31]. Nutrients of N, P_2_O_5_, and K_2_O were delivered using qualitative proportions of 10%, 7%, and 9%, respectively. Fertilizers were delivered through 16 applications once a week over four months.

**Fig 1:**
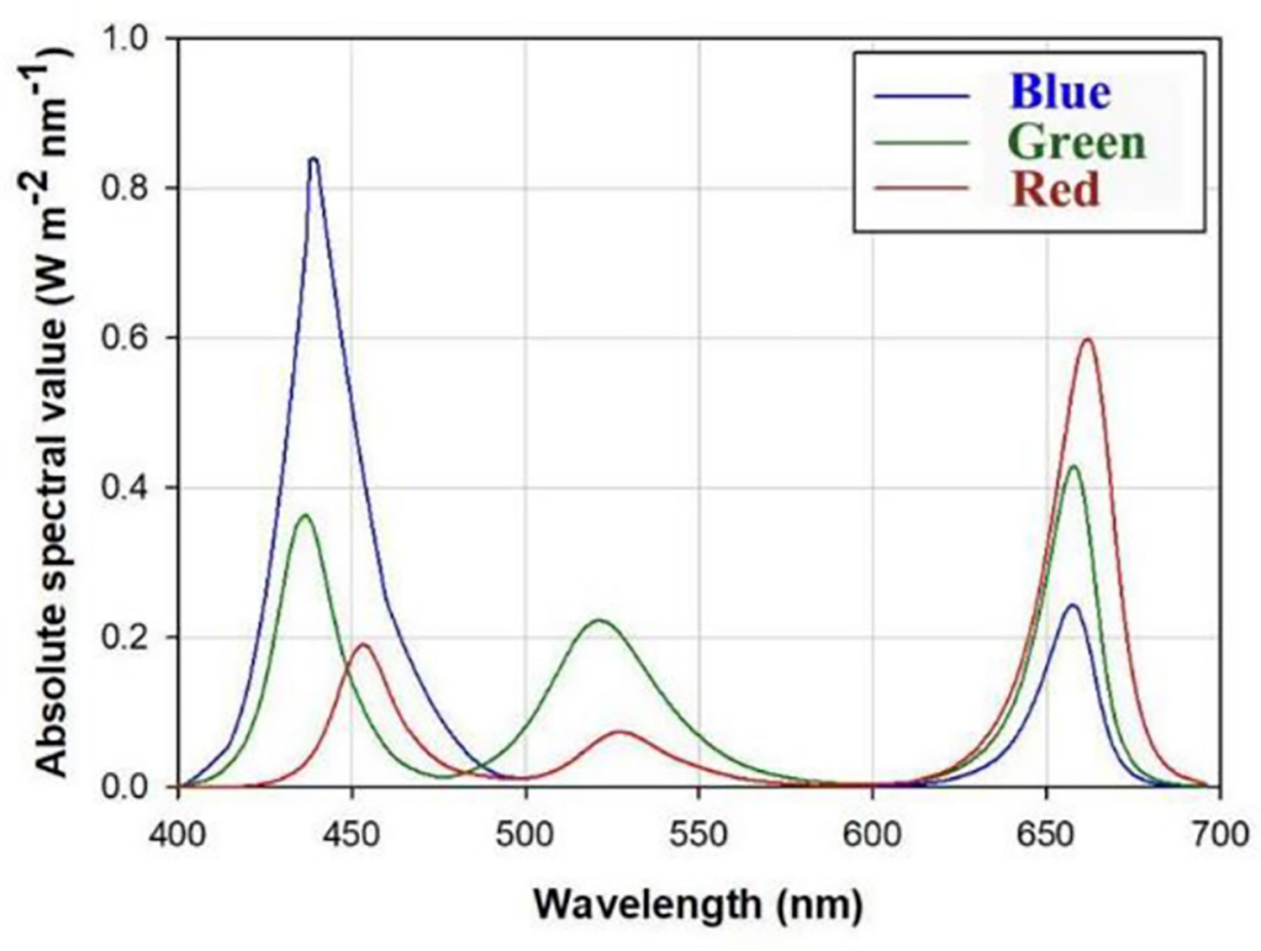
Absolute spectral values in accordance with wavelengths between 400nm and 700nm for three types of light-emitting diode (LED) lighting spectra from red-, green-, and blue-lights.

Seedlings were raised under LED panels (each: 0.5m width and 1.2m length) (Pudao Photoelectricity, Changchun, China) which supplied three types of illuminating spectra. Electric flow for every diode was adjusted by an electrical transformer. The red-light power was 200 W and the green- and blue-lights’ powers were 135 W. Electric current flow from each transformer was regulated to vary combined emitting spectra. Specific optical characteristics of three spectra are shown in Table 1. Absolute spectral values in accordance with wavelengths between 400–700 nm for three types of spectra are shown in Fig 1. Seedlings were subjected to an 18-h daily photoperiod (06:00 am to 24:00 pm) [8, 12]. Tanked seedlings were assigned as a basic sampling unit and six replicates of tanks were assigned for one combined spectra and Cos treatment. Temperature ranged between 28°C and 38°C while relative humidity was set to be 85±1%.

**Table 1:** The quality of spectra 40 cm beneath the light-emitting diode (LED) panel for three types of LED lights.

Light source		PPFD^1^ (μmol m^-2^ s^-1^)			
		Total	Red (%)	Green (%)	Blue (%)
Blue	10% R, 10% G, 70% B ^2^	95.18	17.8	33.7	48.5
Green	20% R, 10%, G, 30% B	96.46	26.2	56.4	17.4
Red	30%, R, 10%, G, 20% B	97.86	71.7	13.7	14.6

Note

^1^ PPFD, photosynthetic photon flux rate

^2^ R, G, B, red-, blue-, and green-light proportions in PPFD.

### Seedling sampling and measurements

When the experiment was nearing its end, seedlings were moved out of pots and divided into parts of shoot and root. All 12 seedlings per tank were used to measure height, RCD, and fine root morphology. Six were randomly chosen and measured for leaf attributes and dry biomass using dried samples. Others were used to measure chlorophyll and leaf protein contents. Samples were oven-dried at 70°C for 72 h and used to quantify dry weight [9, 35]. After passing 1-mm sieves, dried samples were ground and used to determine N and P concentrations. A 0.2-g sample was digested in sulfuric acid, diluted, and analyzed by the Kjeldahl method. P concentration was measured using Inductively Coupled Plasma Optical Emission Spectrometry (Thermo Fisher Scientific, Waltham, MA, USA) [36].

Quality was evaluated by the classic model [37]:

$$DQI=\frac{B_{W}}{\frac{H}{D}+\frac{B_{S}}{B_{R}}}$$ (1)

where *DQI* is the assessed seedling quality index; *B*_*W*_ is the biomass of whole plant; *B*_*S*_ and *B*_*R*_ are biomass in parts of shoot and root, respectively; *H* and *D* are seedling shoot height and RCD, respectively. Nutrient (N or P) utilization index (*NUI* or *PUI*) was evaluated as the evaluation of photosynthetic production per unit leaf nutrient mass [19]:

$$NUI/PUI=\frac{{Bio}_{Whole}}{\%{N/P}_{Shoot}}$$ (2)

where %*N/P*_*Shoot*_ is the percent N or P concentration in shoot as percentage for calculating *NUI* or *PUI*, respectively. Efficiencies of N and P uptakes (i.e., *NUE* and *PUE*, respectively) were evaluated by a modified model that was used by Chu et al. [38]:

$$NUE/PUE=\frac{{Bio}_{Whole}\times {\%N/P}_{Whole}}{{Root}_{Fine}}$$ (3)

where, %*N/P*_*Whole*_ is percent N or P concentrations in whole plant for *NUE* or *PUE*, respectively. *Root*_*Fine*_ can be either fine root length, surface-area, or tip-number.

Eight needlelike leaves were randomly sampled from one seedling and sent to be scanned. Fine roots were rinsed and carefully excised and also used for scanning. Both leaves and roots were scanned to obtain digital images. Scanned root images were analyzed by WinRHIZO software (Regent Ltd., Alberta, Canada) to measure fine root length, surface area, and tip number. Scanned leaf images were further processed as a histogram. The green-color channel was used to quantify green color (*GCI*). Leaf area (LA) can be calculated by the following model:

$$LA=\frac{{Pixel}_{A}}{{Rs.}^{2}\times {Leaf}_{N}}$$ (4)

where *Pixel*_*A*_ is the whole pixel value for the projected area; *Rs*. is the resolution; *Leaf*_*N*_ is leaves’ number.

Leaf concentrations of photosynthetic pigments and soluble protein were determined according to the method of Zhao et al. [21]. The wavelength for measuring chlorophyll-a, chlorophyll-b, and carotenoid were measured at 663 nm, 645 nm, and 470 nm, respectively. Soluble protein was measured at 650 nm.

### Statistical analysis

We analyzed data using SPSS software. Data were tested for normality, and all passed. Cos spray and spectral condition on seedling parameters were assigned as two fixed factors which were input to a two-way analysis of variance (ANOVA) model as independent variables to detect their interaction on seedling parameters. Significant results were averaged for means and ranked and compared by Tukey test (α = 0.05). Vector diagnosis was used to evaluate nutritional states in the shoot part [39]. Pearson analysis was used to detect the correlation between assessed quality and nutrient utilization.

## Results

### Aerial part growth

**Fig 2:**
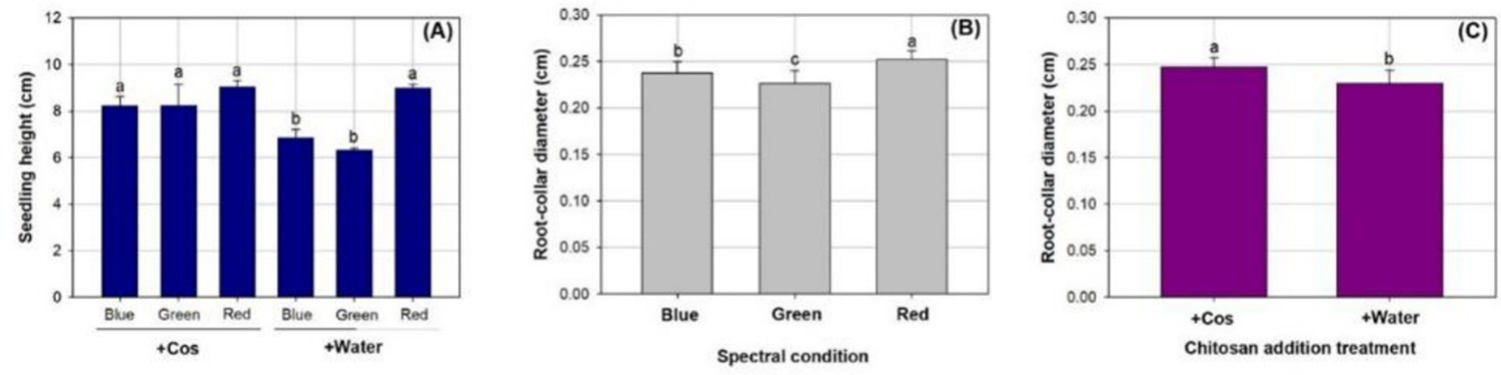
Shoot height (A) and root-collar diameter (B and C) in *Podocarpus macrophyllus* seedlings exposed to chitosan (+Cos) or water (+Water) sprays in three types of light emitting diodes spectra in red, green, and blue lights. Bar height corresponds to the mean of the measurement while the error bars represent standard errors. Different letters represent difference at significant level of 0.05 by Tukey test.

Spectral condition and Cos spray had an interactive effect on seedling height (Table 2). Either of the factors showed a significant main effect on RCD, but no interactive effect was detectable. Seedlings sprayed by water in blue and green light spectra had lower height than seedlings in other treatments (Fig 2A). RCD was the highest in the red-light spectrum (0.25±0.01 cm), followed by that in the blue-light spectrum (0.24±0.01 cm), and was lowest in the green-light spectrum (0.23±0.01 cm) (Fig 2B). In addition, seedlings exposed to Cos spray had greater RCD (0.25±0.01 cm) than those exposed to water spray (0.23±0.01 cm) (Fig 2C).

**Table 2:** Analysis of variance (ANOVA) of spectral condition (SC), oligosaccharide spray (OA), and their combination (SC×OA) on morphology, dry weight biomass, and quality assessment in *Podocarpus macrophyllus* seedlings.

Seedling parameters	Source of variation					
	SC		OA		SC×OA	
	*F*	*Sig*.	*F*	*Sig*.	*F*	*Sig*.
Height	**42.47** ^1^	**<0.0001**	**45.85**	**<0.0001**	**11.64**	**0.0002**
RCD ^2^	**19.40**	**<0.0001**	**27.46**	**<0.0001**	1.40	0.2622
FRL ^3^	**8.56**	**0.0011**	**24.48**	**<0.0001**	0.05	0.9554
FRS ^4^	**6.48**	**0.0046**	**21.28**	**<0.0001**	0.04	0.9595
FRT ^5^	**7.38**	**0.0025**	**23.83**	**<0.0001**	0.32	0.7314
Shoot dry weight	**16.37**	**<0.0001**	**10.12**	**0.0034**	**19.00**	**<0.0001**
Root dry weight	**12.58**	**0.0001**	**8.21**	**0.0075**	**25.79**	**<0.0001**
Whole-plant dry weight	**20.40**	**<0.0001**	**13.73**	**0.0009**	**28.20**	**<0.0001**
R/S ^6^	2.22	0.1264	0.12	0.7300	1.38	0.2682
*DQI* ^7^	**15.86**	**<0.0001**	**11.38**	**0.0021**	**26.65**	**<0.0001**

Note

^1^ Significant values in bold

^2^ RCD abbreviates root-collar diameter

^3^ FRL abbreviates fine root length

^4^ FRS abbreviates fine root surface-area

^5^ FRT abbreviates fine root tip-number

^6^ R/S abbreviates dry weight ratio of root to shoot parts

^7^ *DQI* abbreviates Dickson quality index.

### Fine root morphology

**Fig 3:**
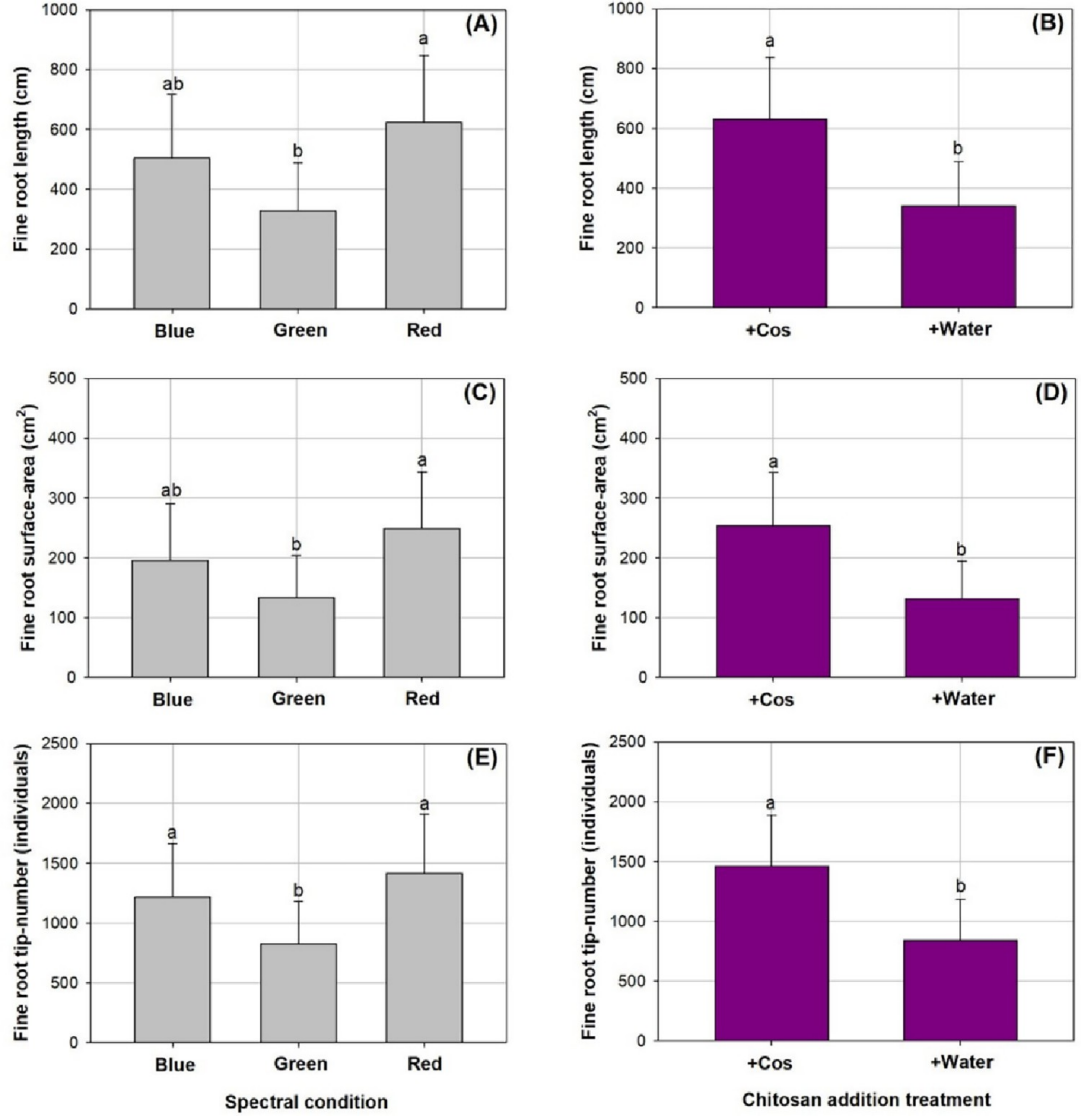
Fine root length (A and B), surface-area (C and D), and tip-number (E and F) in *Podocarpus macrophyllus* seedlings exposed to main effects of chitosan (+Cos) and water (+Water) sprays and the variation among three types of light emitting diodes spectra in red, green, and blue lights. Bar height corresponds to the mean of the measurement while the error bars represent standard errors. Different letters represent difference at significant level of 0.05 by Tukey test.

None of the fine root parameters showed significant response to the combined spectral and Cos treatments (Table 2). However, both main effects of spectral condition and Cos spraying were significant for all three assessed fine root parameters (Fig 3). The spectrum of red-color light resulted in longer fine roots (624.43±222.83 cm) compared to the green-light spectrum (328.22±161.06 cm), but both were not statistically different compared to the blue-light spectrum (504.47±211.82 cm) (Fig 3A). Fine root surface was also larger in the red-light spectrum (249.42±94.04 cm^2^) compared to that in the green-light spectrum (133.08±70.63 cm^2^) with no difference from that in the blue-light spectrum (195.64±95.07 cm^2^) (Fig 3C). Root tip-number was lower in the green-light spectrum (825.58±355.51 individuals) relative to that in the red- (1416.25±492.20 individuals) and blue-light spectrums (1218.67±445.97 individuals) (Fig 3E). Compared to seedlings receiving water spray, those exposed to the Cos spray had greater length (340.21±148.97 cm and 631.21±206.28 cm, respectively) (Fig 3B), surface-area (253.64±89.13 cm^2^ and 131.79±62.95 cm^2^, respectively) (Fig 3D), and tip-number in fine roots (841.61±343.29 individuals and 1465±419.64 individuals, respectively) by 86%, 92%, and 74%, respectively (Fig 3F).

### Dry weight and quality assessment

**Fig 4:**
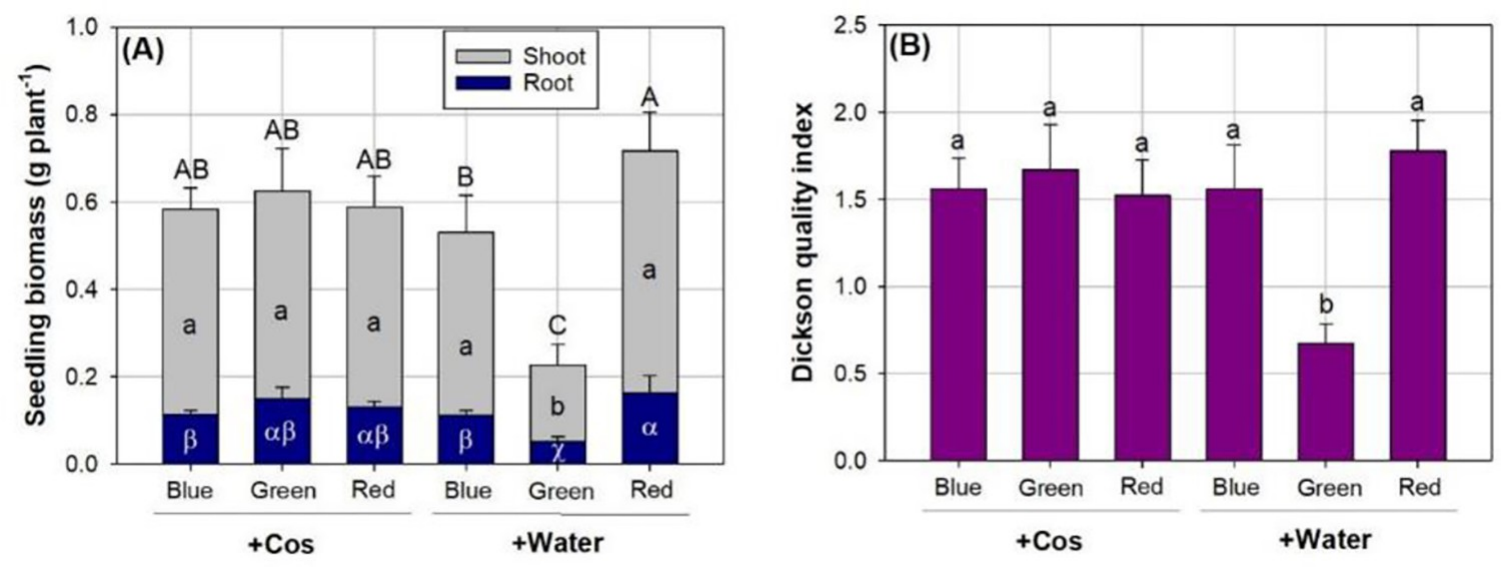
Shoot and root parts’ biomass (A) and Dickson quality index (B) in *Podocarpus macrophyllus* seedlings exposed to chitosan additive (+Cos) and water (+Water) sprays in three types of light emitting diodes spectra from red, green, and blue lights (*n* = 6). Bar height corresponds to the mean of the measurement while the error bars represent standard errors. Different letters represent difference at significant level of 0.05 by Tukey test. In cell A, capital letters label the difference of whole-plant biomass, lower-case-letters label the difference for shoot, and Greek alphabet letters label difference of biomass in root.

Dry weight was significantly responsive to the combined effects of spectral condition and Cos spray in parts of shoot and root and in whole plant (Table 2). Biomass in shoots was lower in water-sprayed seedlings in the spectrum of green light compared to those in combined treatments (Fig 4A). Root biomass was also lowest in seedlings exposed to a combination of green-light and water-spray treatment. Seedlings exposed to red light with water spray had more abundant root biomass than those in the blue light with either water or Cos sprays. As a result, biomass in whole plant was greater in seedlings receiving water spray in the red-light spectrum than those receiving water spray in the green and blue light spectra. *DQI* was found to be lower for seedlings with water spray in green light compared to those subjected to combined red-light and water-spray treatment (Fig 4B). The interaction between green-light spectrum and Cos spray also resulted in a higher *DQI* than that in combined treatments.

### Concentrations of N and P

**Fig 5:**
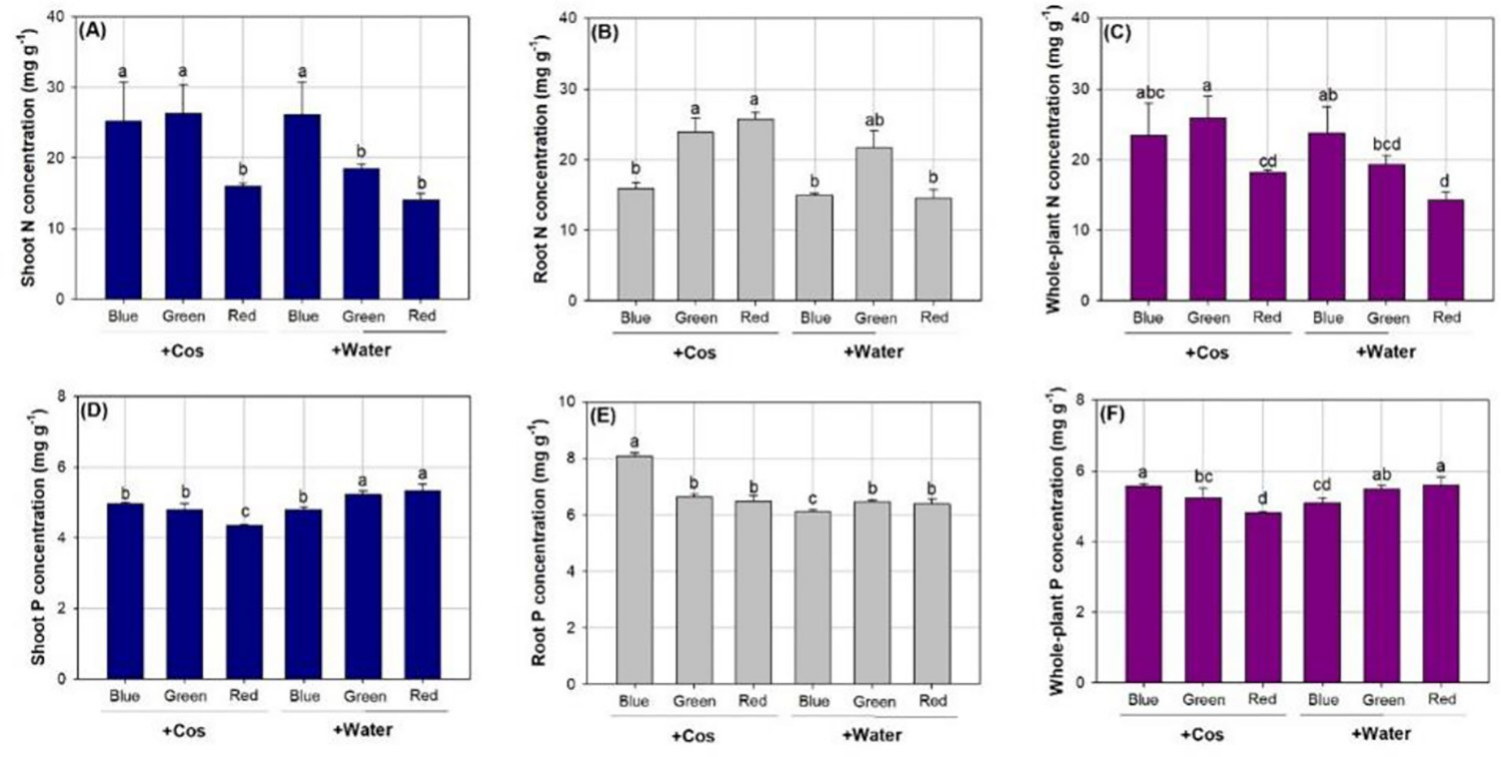
Concentrations of nitrogen (N) (A, B, and C) and phosphorus (P) (D, E, and F) in parts of shoot (left), root (middle), and whole plant (right) in *Podocarpus macrophyllus* seedlings exposed to chitosan (+Cos) or water (+Water) sprays in three types of light-emitting diodes spectra from red, green, and blue lights. Bar height corresponds to the mean of the measurement while the error bars represent standard errors. Different letters represent difference at significant level of 0.05 by Tukey test.

Combined spectrum and Cos treatments showed a significant effect on concentrations of N and P (Table 3). Seedlings exposed to the spectrum of blue light with both Cos and water sprays had higher N concentration in shoots than those in the spectrum of red light (Fig 5A). In the spectrum of green light, seedlings with Cos spray also showed higher N concentration in shoots than those with water spray. Root N concentration was higher in seedlings subjected to the green-light spectrum with Cos spray than those subjected to the blue-light spectrum (Fig 5B). In the red-light spectrum, seedlings with Cos spray had higher root N concentration than those with water spray. N concentration in whole plant was higher in seedlings exposed to the spectrum of green light with Cos spraying relative to those with water spraying or in the red-light spectrum (Fig 5C).

**Table 3:** ANOVA of spectral condition (SC), oligosaccharide spray (OA), and their combination (SC×OA) on concentrations (conc.) and contents (cont.) of nitrogen (N) and phosphorus (P) in *Podocarpus macrophyllus* seedlings.

Seedling parameters	Source of variation					
	SC		OA		SC×OA	
	*F*	*Sig*.	*F*	*Sig*.	*F*	*Sig*.
Shoot N conc.	**25.46**	**<0.0001**	**5.65**	**0.0240**	**4.18**	**0.0250**
Root N conc.	**8.68**	**0.0011**	**10.63**	**0.0028**	**4.96**	**0.0138**
Whole-plant N conc.	**20.96**	**<0.0001**	**11.25**	**0.0022**	**4.12**	**0.0262**
Shoot P conc.	**4.96**	**0.0138**	**86.34**	**<0.0001**	**53.69**	**<0.0001**
Root P conc.	**63.92**	**<0.0001**	**219.08**	**<0.0001**	**146.63**	**<0.0001**
Whole-plant P conc.	2.63	0.0890	**9.18**	**0.0050**	**37.24**	**<0.0001**
Shoot N cont.	**7.90**	**0.0017**	**12.90**	**0.0012**	**11.94**	**0.0002**
Root N cont.	**8.52**	**0.0012**	**28.62**	**<0.0001**	**9.05**	**0.0008**
Whole-plant N cont.	**4.52**	**0.0193**	**23.05**	**<0.0001**	**16.47**	**<0.0001**
Shoot P cont.	**13.69**	**<0.0001**	2.66	0.1134	**22.69**	**<0.0001**
Root P cont.	**10.01**	**0.0005**	**22.29**	**<0.0001**	**24.56**	**<0.0001**
Whole-plant P cont.	**20.36**	**<0.0001**	**9.66**	**0.0041**	**37.38**	**<0.0001**

P concentration in the shoot part was higher in seedlings exposed to the spectra in green and red lights with water spraying compared to those in other treatments (Fig 5D). Seedlings in the red-light spectrum with Cos spray had the lowest P concentration in the shoot part. Among all combined treatments, P concentration in the root part was highest in the blue-light spectrum with Cos spray but seedlings in the blue-light spectrum with water spray had the lowest P concentration in the root part (Fig 5E). P concentration in whole plant was the highest in seedlings exposed to the spectrum of blue light with Cos spraying and those in the spectrum of red light with water spraying (Fig 5F). Seedlings in the red-light spectrum with Cos spray showed the lowest whole-plant P concentration.

### Contents of N and P

**Fig 6:**
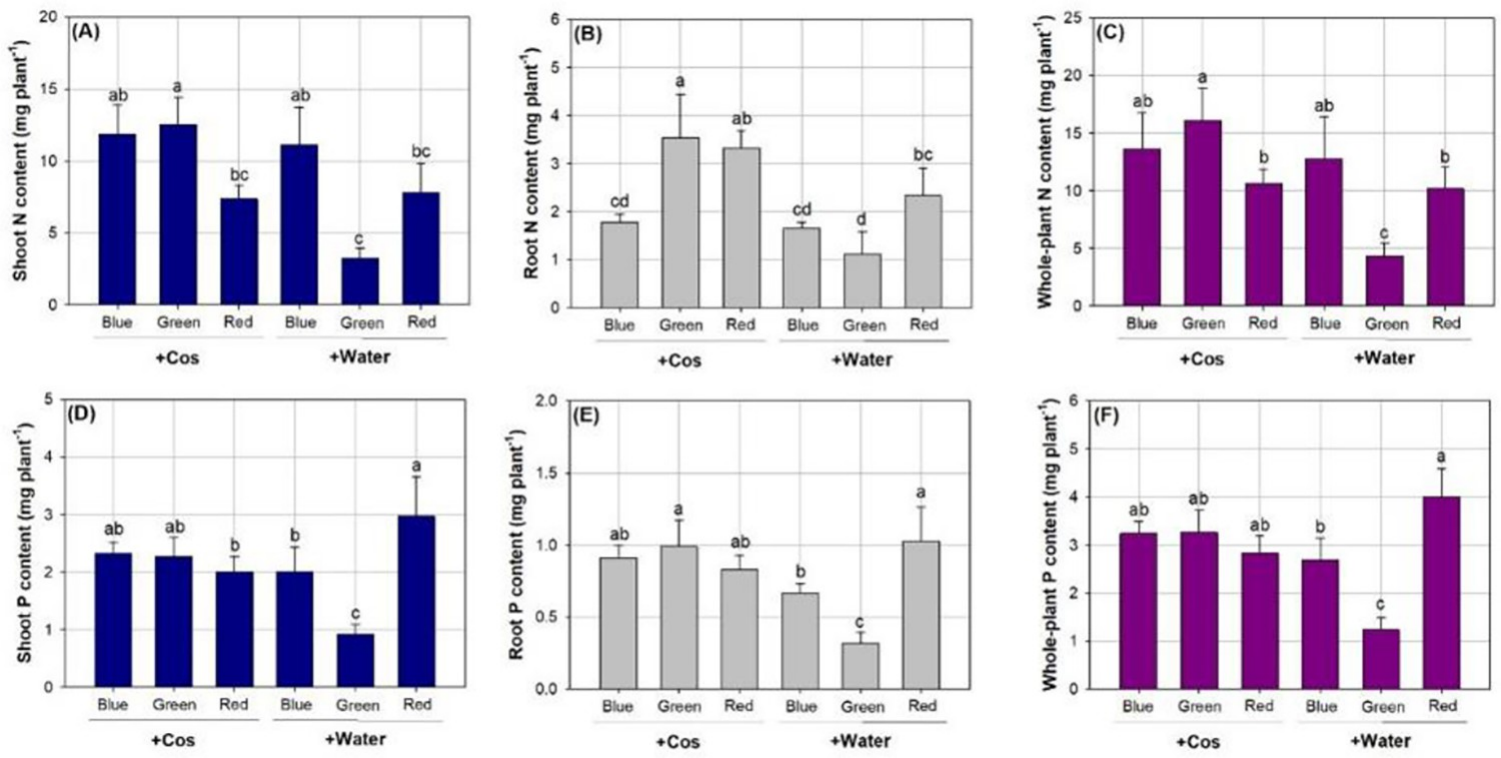
Contents of nitrogen (N) (A, B, and C) and phosphorus (P) (D, E, and F) in shoot (left), root (middle), and whole plant (right) in *Podocarpus macrophyllus* seedlings exposed to chitosan oligosaccharide (+Cos) or water (+Water) sprays in three types of light-emitting diodes spectra from red, green, and blue lights. Bar height corresponds to the mean of the measurement while the error bars represent standard errors. Different letters represent difference at significant level of 0.05 by Tukey test.

Combined spectra and Cos treatments had a significant effect on contents of N and P (Table 3). N content in shoot was lower in seedlings exposed to the spectrum of green light than to the spectrum of red light with either Cos or water spraying (Fig 6A). Seedlings receiving Cos spray in the spectrum of green light showed higher root N content than seedlings in most other treatments except for those subjected to the red-light spectrum and Cos spray (Fig 6B). As a result, N content in whole plant was also higher in seedlings subjected to the spectrum of green light with Cos spraying relative to those subjected to the spectra of green- and red-lights with water spraying (Fig 6C).

P content in shoot part was higher in seedlings exposed to the spectrum of red light with water spraying than those exposed to the spectrum of red light with Cos spraying and those in the spectra of blue- and green-lights with water spraying (Fig 6D). Again, seedlings in the spectrum of red light with water spraying showed higher P content in the root part relative to those receiving water spray in spectra of blue and green lights (Fig 6E). Finally, the whole-plant P content was highest in water-treated seedlings subjected to the spectrum of red light relative to those exposed to the spectra of blue and green lights (Fig 6F).

### Foliar parameters

Spectral condition and Cos spray had a combined effect on LA (*F*_2,30_ = 12.32; *Sig*. = 0.0001), single leaf weight (*F*_2,30_ = 3.79; *Sig*. = 0.0340), SLA (*F*_2,30_ = 11.89; *Sig*. < 0.0001), and *GCI* (*F*_2,30_ = 3.75; *Sig*. = 0.0352). Seedlings in the spectrum of red light with Cos spraying had larger LA than those sprayed by water in spectra of blue and green lights (Table 4). Single leaf weight was higher in seedlings exposed to the spectrum of red light with Cos spray than in those exposed to spectra of blue and green lights. In contrast, SLA was found to be lower in seedlings in the spectra of red and green lights with Cos spray. *GCI* was found to be higher in seedlings in the spectrum of red light compared to seedlings subjected to other treatments.

**Table 4:** Foliar parameters in *Podocarpus macrophyllus* seedlings subjected to combined treatments with blue, green, and red spectra and chitosan or water sprays (+Cos vs +Water) (*n* = 6).

Foliar parameter	Blue		Green		Red	
	+Cos	+Water	+Cos	+Water	+Cos	+Water
Area (cm^-2^)	640.0±231.7ab	369.0±123.5bc	485.3±92.7ab	171.1±55.3c	768.3±242.7a	480.5±129.0abc
Weight (mg)	13.89±0.60cd	12.30±1.21d	15.66±0.67bc	11.68±1.13d	18.64±2.14a	17.33±0.66ab
SLA ^1^	0.25±0.01bc	0.29±0.03a	0.21±0.01c	0.27±0.03ab	0.21±0.04c	0.28±0.01ab
*GCI* ^2^	92.78±1.62b	99.62±2.39b	97.55±3.93b	95.98±5.51b	109.43±5.01a	108.42±3.07a

Note

^1^ SLA, Specific leaf area

^2^ *GCI*, green color index.

**Fig 7:**
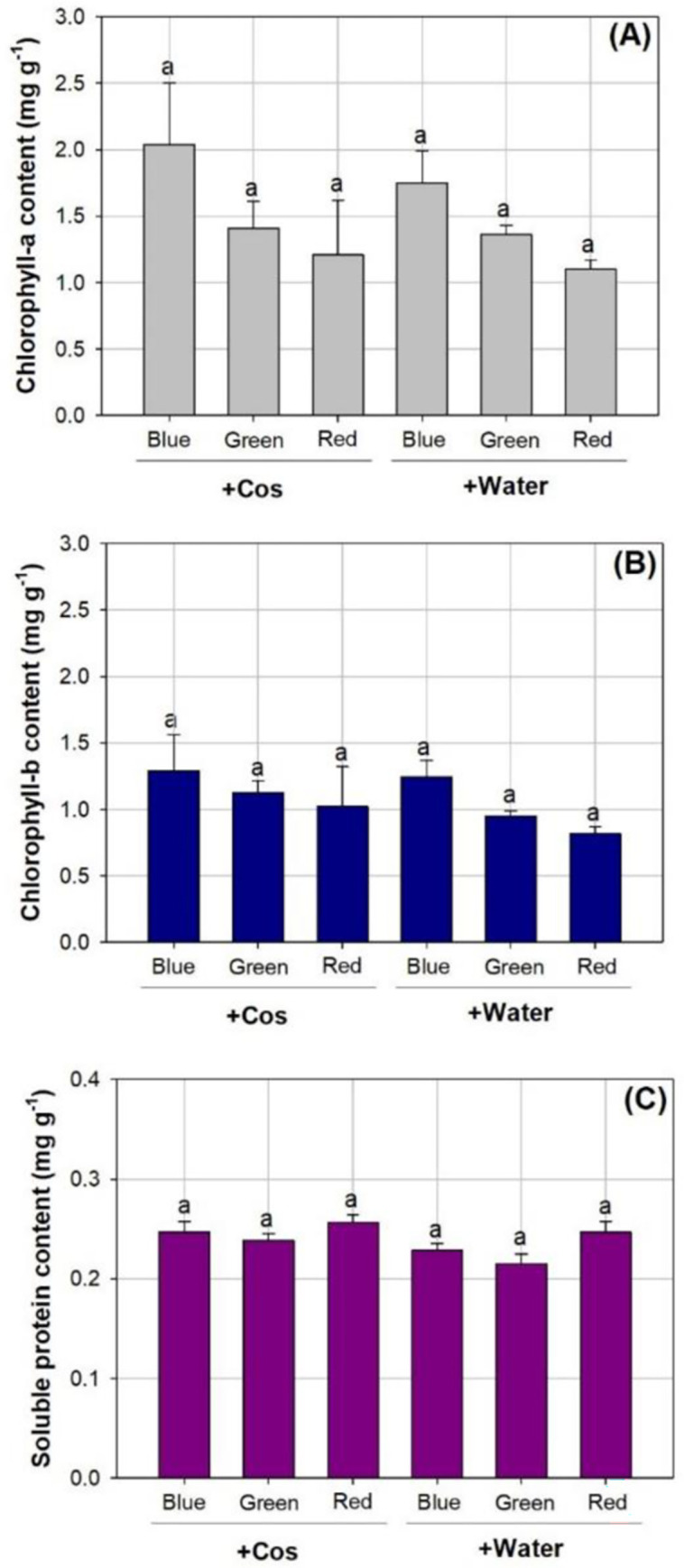
Concentrations in chlorophyll-a (A), chlorophyll-b (B), and soluble protein (C) in *Podocarpus macrophyllus* seedlings subjected to effects of chitosan (+Cos) and water (+Water) sprays and three types of light emitting diodes spectra from red, green, and blue lights. Bar height corresponds to the mean of the measurement while the error bars represent standard errors. Different letters represent difference at significant level of 0.05 by Tukey test.

Spectrum had a significant main effect on concentrations of chlorophyll-a (*F*_2,30_ = 17.94; *Sig*. < 0.0001), chlorophyll-b (*F*_2,30_ = 9.64; *Sig*. = 0.0006), and soluble protein (*F*_2,30_ = 19.40; *Sig*. < 0.0001). The concentration of chlorophyll-a was higher in the spectra of blue light (1.89±0.36 mg g^-1^) than in the spectra of green (1.38±0.14 mg g^-1^) and red lights (1.15±0.31 mg g^-1^) by 37% and 39%, respectively (Fig 7A). However, chlorophyll-a content did not vary between the Cos spray (1.55±0.46 mg g^-1^) and the water spray treatments (1.40±0.27 mg g^-1^). Chlorophyll-b concentration was higher in the blue-light spectrum (1.27±0.20 mg g^-1^) compared to that in the green- (1.04±0.10 mg g^-1^) and red-light spectra (0.92±0.22 mg g^-1^) by 22% and 38%, respectively (Fig 7B). Concentration of chlorophyll-b was higher by 14% with Cos spraying (1.14±0.24 mg g^-1^) than that with water spraying (1.00±0.17 mg g^-1^). Soluble protein concentration was the highest in the red-light spectrum (0.25±0.01 mg g^-1^), followed by that in the blue-light spectrum (0.24±0.01 mg g^-1^), and lowest in the green-light spectrum (0.23±0.01 mg g^-1^) (Fig 7C). Again, concentration of soluble protein was also higher by 7% with Cos spraying (0.25±0.01 mg g^-1^) compared to that with water spraying (0.23±0.01 mg g^-1^).

### Uptake and utilization for N and P nutrients

Combined spectra and Cos spray showed a significant effect on *NUI* (*F*_2,30_ = 22.26; *Sig*. < 0.0001) and *PUI* (*F*_2,30_ = 21.97; *Sig*. < 0.0001). *NUI* was highest in seedlings exposed to the spectrum of red light with water spraying, followed by those with Cos spray, and the lowest in water-sprayed seedlings in the blue- or green-light spectra (Table 5). *PUI* was higher in seedlings receiving Cos spray in the green- and red-light spectra and seedlings receiving water spray in the red-light spectrum (Table 5).

**Table 5:** Assessments of uptakes and utilizations in nitrogen (N) and phosphorus (P) nutrients in *Podocarpus macrophyllus* seedlings in response to combined spectra (Blue, Green, and Red) and chitosan and water sprays (+Cos vs +Water) (*n* = 6).

Efficiency variables	Blue		Green		Red	
	+Cos	+Water	+Cos	+Water	+Cos	+Water
*NUI* ^1^	2.41±0.51c	2.07±0.47cd	2.42±0.48c	1.22±0.26d	3.67±0.49b	5.10±0.45a
*PUI* ^2^	11.76±1.05ab	11.02±1.80ab	13.09±2.50a	4.32±0.97b	13.48±1.60a	13.39±1.38a
NUFRL ^3^	0.33±0.17a		0.33±0.12a		0.19±0.08b	
NUFRS ^4^	0.91±0.56a		0.83±0.35a		0.48±0.18b	
PUFRL ^5^	7.14±3.15a		7.72±2.91a		6.56±3.19a	
PUFRS ^6^	19.78±10.46a		19.60±7.92a		16.44±7.72a	

Note

^1^ *NUI* stands for N utilization index

^2^ *PUI* stands for P utilization index

^3^ NUFRL, N uptake per fine root length

^4^ NUFRS, N uptake per fine root surface-area

^5^ PUFRL, P uptake per fine root length

^6^ PUFRS, P utilization per fine root surface-area.

The Cos spraying treatment did not have any effects on NUFRL, NUFRS, PUFRL, and PUFRS, nor did it interact with spectral condition. Therefore, the spectral condition treatment showed a main effect on these parameters. The red-light spectrum resulted in lower NUFRL (*F*_2,30_ = 3.56; *P* = 0.0409) and NUFRS (*F*_2,30_ = 3.34; *P* = 0.0489) compared to the blue- and green-light spectra (Table 5). However, the spectral condition treatment had no effect on PUFRL and PUFRS.

### Pearson correlation

As is shown in Table 6, *DQI* had no correlation with *NUI* but had a positive relationship with *PUI*. Any of NUFRL, NUFRS, and NUFRT were not correlated to *DQI*. Instead, a positive relationship was found between any two of these three parameters.

**Table 6:** Pearson correlation among pairs of uptakes and utilizations in nitrogen (N) and phosphorus (P) nutrients in *Podocarpus macrophyllus* seedlings.

Variables	Index	*DQI*	*NUI*	*PUI*	NUFRL	NUFRS	NUFRT
*DQI* ^1^	*F*	1	0.6753	0.9627 ^2^	-0.1008	-0.0441	0.1176
	*Sig*.	-	0.1411	0.0021	0.8493	0.9338	0.8244
*NUI* ^3^	*F*	-	1	0.71381	-0.5955	-0.5585	-0.4088
	*Sig*.	-	-	0.1111	0.2124	0.2494	0.421
*PUI* ^4^	*F*	-	-	1	-0.29382	-0.2382	-0.1049
	*Sig*.	-	-	-	0.572	0.6495	0.8433
NUFRL ^5^	*F*	-	-	-	1	0.9872	0.9639
	*Sig*.	-	-	-	-	0.0002	0.0019
NUFRS ^6^	*F*	-	-	-	-	1	0.95122
	*Sig*.	-	-	-	-	-	0.0035
NUFRT ^7^	*F*	-	-	-	-	-	1
	*Sig*.	-	-	-	-	-	-

Note

^1^ *NUI*, N utilization index

^2^ Values in gray cells indicate significant correlation

^3^ *PUI*, P utilization index

^4^ NUFRL, N uptake per fine root length

^5^ NUFRS, N uptake per fine root surface-area

^6^ PUFRL, P uptake per fine root length

^7^ PUFRS, P utilization per fine root surface-area.

## Discussion

Our findings on seedling quality did not support our first hypothesis. We did not observe any promotion on seedling quality in response to combined Cos spraying and blue-light spectrum illumination. We consider this failure of Cos effect resulted from the null effect on R/S in our Luohan pine seedlings, as like null effects on seedlings of *Cordyline terminalis* [28] and *D*. *odorifera* [29]. When combined with the blue-light spectrum, Cos spray increased height growth but failed to impose any effect on other parameters that were used for assessing quality assessment. Both Montagnoli et al. [40] and Navidad et al. [16] proved that a spectrum with high blue-light proportion can promote seedling growth and biomass. However, a faster growth speed induced by some spectra does not mean a definite response of elevated seedling quality. It was not the first time in our study finding that Cos addition can remarkably enhance growth and biomass in plants [27, 41]. It was reported that Cos addition promoted seedling quality in *Dalbergia odorifera* seedlings in combination with a spectrum of higher red-light proportion [29]. In our study, the spectrum high in red light only increased seeding quality compared to the green-light spectrum with water spraying. Spectra high in red light vs blue light did not induce any difference of DQI. These findings agree to those found by Smirnakou et al. [12] who revealed that the best seedling quality for *Quercus ithaburensis* var. *macrolepis* was found in the spectrum with the highest red-light proportion. In contrast, it was also found that in *Pinus koraiensis* seedling quality was highest in the spectra with high green light proportion [8]. According to Wendy’s green-depletion experiment [42], green light may depress biomass accumulation relative to red and blue lights. In white-light LED with a higher proportion of green light, Luohan pine seedlings were also reported to have a better quality compared to other spectrum from high-pressure sodium illuminator [7]. Therefore, there may be a species × spectra interaction that imposes significant impacts on seedling quality.

We failed to find any interactive effects between LED spectra and Cos addition on fine root morphology, which agrees with results on *Eleutherococcus senticosus* [14]. The increase of fine root length and surface-area caused by Cos spray was also reported by Guo et al. [14]. However, greater fine root morphology in the high-green spectrum in our study did not occur in *E*. *senticosus* [14], *Bletilla striata* [43], *Ficus hirta*, and *Alpinia oxyphylla* [9]. All these formally published studies reported a larger fine root system in the spectrum enriched in red light. However, it was also reported that *Allium victorialis* sprouts showed no response of fine root morphology to different LED spectra [35]. He et al. [10] reported that *Pinus pumila* places more fine roots in the red-light spectrum, which concurs with our findings. These different responses of fine root morphologies to LED may result from the natural attributes among species. Luohan pine is a heliophile that has a different fine root arrangement compared to shade-tolerant plants [17]. As a dweller of the understory layer, *E*. *senticosus* has acclimated to high shading and more green light proportion in the sunlight spectrum [44]. The plants mentioned were all highly tolerant to shade as well [9, 35, 43]. Juvenile trees, such as Luohan pine and *P*. *pumila* [10], like to place fine roots in soils subjected to spectra high in red light to benefit root foraging.

Relative to spectra of green and blue lights, the spectrum of red light tended to cause relatively lower N richness as increased biomass but decreased concentration. The negative nutrient status in red light has also been reported on *P*. *koraiensis* [8] and *D*. *odorifera* [29] seedlings. Similar to ours, these studies revealed that the red-light spectrum tended to induce N dilution if no additional N was input. This is mainly the result of continuous lighting that promotes biomass production and dilutes N concentration [30]. We cultured Luohan pine seedlings with exponential fertilization at the dose of 60 mg N plant^-1^. This was proven to be the lowest dose to induce luxury consumption [6, 31]. This dose was also obtained from an experiment using white-light LED whose spectrum comprised 17% red, 75% green, and 8% blue lights wavelengths. Therefore, the change of spectral condition from high-green light spectrum to one with high-red light enlarged the demand for N and resulted in negative N status. The switch of Cos addition on N deficiency from the blue-light spectrum to green-light resulted from the significant effect of Cos spray on biomass increase and quality improvement in the green light spectrum.

The interaction of Cos spray and red-light spectrum resulted in P excess, i.e. high P concentration but lowered biomass. This was caused because Cos addition increased P concentration in the red-light spectrum without any promotion on biomass. Excessive P accumulation in the high-red spectrum was also reported on *P*. *Koraiensis* [8] and *A*. *elata* [20] seedlings. Our results also suggest that the Cos spray cannot promote P uptake in the spectrum of green light, leading to P dilution relative to the spectrum of blue light. This occurred because the Cos spray in the spectrum of green light increased biomass without any effect on P uptake. Wang et al. [41] found that Cos spray can mediate P consumption for biomass production in Luohan pine, explaining the biomass production increment in our study. In another study, Dar et al. [45] made a specific experiment of Cos addition to *Trigonella foenum*-*graecum* L and found that Cos hardly increased P uptake unless additional P was added to plants. Future studies on the effect of Cos addition on P metabolism and utilization for biomass production is necessary.

Leaf area was found to have a positive relationship with nearly all biomass and nutrient parameters in Luohan pine seedlings [31, 35]. In our study, leaf area can only partly predict most responses. For example, N and P concentrations were promoted to be the highest in the red-light and Cos combination, which also resulted in high leaf area. Results obtained by Xu et al. [31] were obtained from an experiment with Luohan pine seedlings in response to a range of fertilizers in one unit lighting environment without any exogeneous spray. However, Cos spray decreased SLA in all three spectral conditions, which can be explained by the improvement of photosynthetic efficiency caused by Cos addition lowering SLA. These results agreed with those about shoot and root morphologies and chlorophyll content which are all a result of improved photosynthesis. *GCI* was negatively related with N concentration in leaves [46]. *GCI* cannot be used for precise prediction of N concentration in whole shoots of Luohan pine seedlings [31]. In our study, *GCI* was highest in the spectrum of red light which was generally in accordance with shoot N concentration.

The increase of chlorophyll content by Cos spray in our study was also reported by Xu et al. [47] on *Prunus davidiana* seedlings. This was attributed to the positive effect of Cos addition on abundance of photosynthetic pigments, subsequently enhancing photosynthetic rate and production that benefit growth and dry weight in parts of shoot and root organs. Up-regulation of protein synthesis and promotion of protein abundance is one of the key mechanisms of adding Cos to plants [48, 49]. Abundant protein accumulation by exogeneous Cos input is the necessary precondition for enzyme synthesis to protect plants by overcoming biotic stress. It was reported that chlorophyll content was occasionally increased by the red-light-high LED spectrum in *Pseudotsuga menziesii* and *P*. *engelmannii* seedlings [50]. For *Q*. *ithaburensis* var. *macrolepis* seedlings, chlorophyll content was higher in some spectra with a higher red-light proportion [12]. However, when the red-light proportion was very high in the spectrum of visible light, chlorophyll content failed to continue to respond [8]. Zhao et al. [21] even found no response of chlorophyll content to the change of spectra. More evidence supports our findings that chlorophyll content was higher in the spectrum of blue light relative to that in spectra of red and green lights [11, 51, 52]. For example, Bach and Swiderski [53] reported that chlorophyll content in *Hyacinthus orientalis* plants was promoted by blue light. Our spectra were adapted using a wide bandwidth, which comprised multiple peaks of wavebands. This lighting spectral condition has been used in many studies to test spectral condition on tree seedlings [20, 21, 53]. However, other relevant factors may also be effective in impacting chlorophyl content by different monochromatic wavelengths, such as lighting intensity, time of irradiation, and position of leaves. Further work is needed to obtain more confirmative results using narrow waveband monochromatic lights.

Studies on a wide range of plant species can support our findings concerning higher protein abundance in the red-light spectrum [54]. In contrast, other studies also found that protein content was benefited by the blue-light spectrum [52]. The highest abundance of soluble protein in leaves did not occur in the spectrum with the highest red-light proportion [8]. As was highlighted by Zhang et al. [54] and Meng et al. [52], the ration of red/blue lights may be more important in determining the abundance of protein than monochromatic red or blue lights.

Greater biomass accumulation but lower concentration of N in seedlings exposed to the spectrum of red light resulted in the highest N utilization. The water sprayed seedlings in the spectrum of red light showed the highest utilization of N. This disagreed with former findings on *D*. *odorifera* seedlings, which showed the highest *NUI* in response to a combination of Cos-addition and high-red spectrum [29]. Lower NUFRL and NUFRS in the red-light spectrum resulted from N uptake at a low cost of fine root morphology. In another study, the high-red spectrum was found to promote *NUI* only under conditions with N input with the reference of no N addition [21]. Therefore, insufficient N input led to a lower *NUI* in Cos sprayed seedlings than in water sprayed seedlings as was discussed previously. The null response of *PUI* to Cos spray in the red-light spectrum also disagreed to those found in a previous study [29]. This resulted from P excess in response to the Cos spray in the red-light spectrum. The reasons for higher N and P utilizations in response to the Cos spray in the green-light spectrum were different. For N utilization, the green-Cos combination increased both biomass and N concentration relative to the green-water interaction, generating a higher rate of increase in biomass than in N uptake. For P utilization, the Cos spray resulted in P dilution, which caused a higher *PUI* due to higher biomass and lower P concentration. The positive relationship between *PUI* and *DQI* agrees to those found by Chu et al. [38]. Both studies suggest that seedling quality can be determined by the ability to utilize P for biomass production. Thus, we can accept our second hypothesis.

## Conclusions

In this study, we found a series of results about morphology, dry weight, uptakes and allocations for N and P, foliar physiology, and quality assessment in Luohan pine seedlings exposed to combined treatments of Cos spray and spectral condition. The red-light spectrum led to a promotion of growth, dry mass production, and utilizations for N and P relative to the spectrum of green light with water spraying. However, the Cos spray had an effective combination with the green-light spectrum to induce resistances to N and P deficiencies, enlarge leaf area, and promote assessed quality. Ultimately, the seedling quality synchronized with the utilization of P. Overall, if nutrient utilization is more important than seedling size and biomass for quality assessment, we do recommend the red-light spectrum for producing Luohan pine stocks.

## Supporting information

S1 Data (PDF)
