# Supplementary material for: Quality assessment and nutrient uptake and utilization in Luohan pine (Podocarpus macrophyllus) seedlings raised by chitosan spraying in varied LED spectra
Source: PLoS One. 2022 Apr 28;17(4):e0267632. doi: 10.1371/journal.pone.0267632 (PMC9049360; doi:10.1371/journal.pone.0267632)
Supplement: S1 Data — (PDF) [file pone.0267632.s001.pdf]

1. Seedling height:

Level of Treatment	N	Value	
		Mean	Std Dev
**OligBlue**	**6**	8.23	0.43
**OligGreen**	**6**	8.25	0.98
**OligRed**	**6**	9.02	0.31
**WaterBlue**	**6**	6.85	0.41
**WaterGreen**	**6**	6.33	0.09
**WaterRed**	**6**	8.99	0.17

2. RCD:

Level of Treatment	N	Value	
		Mean	Std Dev
**OligBlue**	**6**	0.25	0.01
**OligGreen**	**6**	0.24	0.01
**OligRed**	**6**	0.26	0.01
**WaterBlue**	**6**	0.23	0.01
**WaterGreen**	**6**	0.22	0.01
**WaterRed**	**6**	0.25	0.01

3. Fine root length:

Level of Treatment	N	Value	
		Mean	Std Dev
**OligBlue**	**6**	639.97	253.44
**OligGreen**	**6**	485.33	101.35
**OligRed**	**6**	768.33	265.44
**WaterBlue**	**6**	368.97	135.12
**WaterGreen**	**6**	171.12	60.51
**WaterRed**	**6**	480.53	141.06

4. Fine root surface area:

Level of Treatment	N	Value	
		Mean	Std Dev
**OligBlue**	**6**	258.01	114.17
**OligGreen**	**6**	197.77	58.42
**OligRed**	**6**	305.14	112.53
**WaterBlue**	**6**	133.27	55.88
**WaterGreen**	**6**	68.40	24.82
**WaterRed**	**6**	193.70	69.48

5. Fine root tip number:

Level of Treatment	N	Value	
		Mean	Std Dev
**OligBlue**	**6**	1460.83	536.52
**OligGreen**	**6**	1187.33	141.64
**OligRed**	**6**	1748.00	550.31
**WaterBlue**	**6**	976.50	363.85
**WaterGreen**	**6**	463.83	149.51

**WaterRed**	**6**	1084.50	341.07

6. Shoot dry mass:

**Level of**	**N**	**Value**	
**Treatment**		**Mean**	**Std Dev**
**OligBlue**	**6**	0.47	0.05
**OligGreen**	**6**	0.48	0.09
**OligRed**	**6**	0.46	0.07
**WaterBlue**	**6**	0.42	0.09
**WaterGreen**	**6**	0.18	0.04
**WaterRed**	**6**	0.56	0.12

7. Root dry mass:

**Level of**	**N**	**Value**	
**Treatment**		**Mean**	**Std Dev**
**OligBlue**	**6**	0.11	0.01
**OligGreen**	**6**	0.15	0.03
**OligRed**	**6**	0.13	0.01
**WaterBlue**	**6**	0.11	0.01
**WaterGreen**	**6**	0.05	0.01
**WaterRed**	**6**	0.16	0.04

8. Shoot N concentration:

**Level of**	**N**	**Value**	
**Treatment**		**Mean**	**Std Dev**
**OligBlue**	**6**	25.18	6.03
**OligGreen**	**6**	26.30	4.51
**OligRed**	**6**	16.03	0.42
**WaterBlue**	**6**	26.10	5.06
**WaterGreen**	**6**	18.53	0.63
**WaterRed**	**6**	14.02	0.98

9. Root N concentration:

**Level of**	**N**	**Value**	
**Treatment**		**Mean**	**Std Dev**
**OligBlue**	**6**	15.85	0.89
**OligGreen**	**6**	23.85	8.76
**OligRed**	**6**	25.78	0.97
**WaterBlue**	**6**	14.95	0.30
**WaterGreen**	**6**	21.68	5.95
**WaterRed**	**6**	14.53	1.36

10. Whole-plant N concentration:

**Level of**	**N**	**Value**	
**Treatment**		**Mean**	**Std Dev**
**OligBlue**	**6**	23.38	4.97
**OligGreen**	**6**	25.87	3.29
**OligRed**	**6**	18.18	0.40

**WaterBlue**	**6**	23.82	4.03
**WaterGreen**	**6**	19.25	1.41
**WaterRed**	**6**	14.17	1.27

11. Shoot P concentration:

**Level of**	**N**	**Value**	
**Treatment**		**Mean**	**Std Dev**
**OligBlue**	**6**	4.95	0.04
**OligGreen**	**6**	4.79	0.20
**OligRed**	**6**	4.36	0.03
**WaterBlue**	**6**	4.79	0.07
**WaterGreen**	**6**	5.22	0.11
**WaterRed**	**6**	5.33	0.22

12. Root P concentration:

**Level of**	**N**	**Value**	
**Treatment**		**Mean**	**Std Dev**
**OligBlue**	**6**	8.06	0.14
**OligGreen**	**6**	6.63	0.12
**OligRed**	**6**	6.47	0.23
**WaterBlue**	**6**	6.10	0.08
**WaterGreen**	**6**	6.45	0.07
**WaterRed**	**6**	6.38	0.20

13. Whole-plant P concentration:

**Level of**	**N**	**Value**	
**Treatment**		**Mean**	**Std Dev**
**OligBlue**	**6**	5.56	0.06
**OligGreen**	**6**	5.24	0.29
**OligRed**	**6**	4.82	0.04
**WaterBlue**	**6**	5.08	0.15
**WaterGreen**	**6**	5.49	0.11
**WaterRed**	**6**	5.59	0.26

14. Shoot N content:

**Level of**	**N**	**Value**	
**Treatment**		**Mean**	**Std Dev**
**OligBlue**	**6**	11.87	3.30
**OligGreen**	**6**	12.55	3.15
**OligRed**	**6**	7.37	1.02
**WaterBlue**	**6**	11.12	3.95
**WaterGreen**	**6**	3.25	0.77
**WaterRed**	**6**	7.83	2.16

15. Roto N content:

**Level of**	**N**	**Value**	
**Treatment**		**Mean**	**Std Dev**
**OligBlue**	**6**	1.78	0.18

**OligGreen**	**6**	3.53	1.33
**OligRed**	**6**	3.33	0.40
**WaterBlue**	**6**	1.65	0.14
**WaterGreen**	**6**	1.12	0.52
**WaterRed**	**6**	2.33	0.63

16. Whole-plant N content:

**Level of**	**N**	**Value**	
**Treatment**		**Mean**	**Std Dev**
**OligBlue**	**6**	13.65	3.45
**OligGreen**	**6**	16.08	3.05
**OligRed**	**6**	10.65	1.37
**WaterBlue**	**6**	12.77	3.99
**WaterGreen**	**6**	4.37	1.20
**WaterRed**	**6**	10.18	2.01

17. Shoot P content:

**Level of**	**N**	**Value**	
**Treatment**		**Mean**	**Std Dev**
**OligBlue**	**6**	2.32	0.22
**OligGreen**	**6**	2.27	0.37
**OligRed**	**6**	2.00	0.30
**WaterBlue**	**6**	2.01	0.46
**WaterGreen**	**6**	0.92	0.20
**WaterRed**	**6**	2.97	0.74

18. Root P content:

**Level of**	**N**	**Value**	
**Treatment**		**Mean**	**Std Dev**
**OligBlue**	**6**	0.91	0.09
**OligGreen**	**6**	0.99	0.20
**OligRed**	**6**	0.83	0.10
**WaterBlue**	**6**	0.67	0.07
**WaterGreen**	**6**	0.32	0.08
**WaterRed**	**6**	1.03	0.26

19. Whole-plant P content:

**Level of**	**N**	**Value**	
**Treatment**		**Mean**	**Std Dev**
**OligBlue**	**6**	3.24	0.28
**OligGreen**	**6**	3.26	0.51
**OligRed**	**6**	2.83	0.40
**WaterBlue**	**6**	2.69	0.49
**WaterGreen**	**6**	1.23	0.27
**WaterRed**	**6**	4.00	0.64

20. Foliar area:

**Level of**	**N**	**Value**

Treatment		Mean	Std Dev
**OligBlue**	**6**	639.97	253.44
**OligGreen**	**6**	485.33	101.35
**OligRed**	**6**	768.33	265.44
**WaterBlue**	**6**	368.97	135.12
**WaterGreen**	**6**	171.12	60.51
**WaterRed**	**6**	480.53	141.06

21. Foliar weight:

Level of	N	Value	
Treatment		Mean	Std Dev
**OligBlue**	**6**	13.89	0.66
**OligGreen**	**6**	15.66	0.74
**OligRed**	**6**	18.64	2.34
**WaterBlue**	**6**	12.30	1.32
**WaterGreen**	**6**	11.68	1.23
**WaterRed**	**6**	17.33	0.73

22. SLA:

Level of	N	Value	
Treatment		Mean	Std Dev
**OligBlue**	**6**	0.25	0.01
**OligGreen**	**6**	0.21	0.01
**OligRed**	**6**	0.21	0.04
**WaterBlue**	**6**	0.29	0.03
**WaterGreen**	**6**	0.27	0.03
**WaterRed**	**6**	0.28	0.01

23. GI:

Level of	N	Value	
Treatment		Mean	Std Dev
**OligBlue**	**6**	92.78	1.77
**OligGreen**	**6**	97.55	4.30
**OligRed**	**6**	109.43	5.48
**WaterBlue**	**6**	99.62	2.61
**WaterGreen**	**6**	95.98	6.03
**WaterRed**	**6**	108.42	3.36

24. Chlorophyl-a:

Level of	N	Value	
Treatment		Mean	Std Dev
**OligBlue**	**6**	2.04	0.50
**OligGreen**	**6**	1.41	0.22
**OligRed**	**6**	1.21	0.45
**WaterBlue**	**6**	1.75	0.26
**WaterGreen**	**6**	1.36	0.07
**WaterRed**	**6**	1.10	0.07

25. Chlorophyl-b:

Level of Treatment	N	Value	
		Mean	Std Dev
**OligBlue**	**6**	1.29	0.30
**OligGreen**	**6**	1.13	0.10
**OligRed**	**6**	1.02	0.33
**WaterBlue**	**6**	1.24	0.14
**WaterGreen**	**6**	0.95	0.04
**WaterRed**	**6**	0.82	0.06

26. Soluble protein:

Level of Treatment	N	Value	
		Mean	Std Dev
**OligBlue**	**6**	0.25	0.01
**OligGreen**	**6**	0.24	0.01
**OligRed**	**6**	0.26	0.01
**WaterBlue**	**6**	0.23	0.01
**WaterGreen**	**6**	0.22	0.01
**WaterRed**	**6**	0.25	0.01
